# High prevalence of protein C, protein S, antithrombin deficiency, and Factor V Leiden mutation as a cause of hereditary thrombophilia in patients of venous thromboembolism and cerebrovascular accident

**DOI:** 10.12669/pjms.306.5878

**Published:** 2014

**Authors:** Nadir Ali, Muhammad Ayyub, Saleem Ahmed Khan

**Affiliations:** 1 Dr. Nadir Ali, FCPS, PhD, Consultant Haematologist, Armed Forces Institute of Pathology (AFIP), Rawalpindi, Pakistan.; 2 Professor Muhammad Ayyub, FRCPath, PhD (London), Professor of Pathology and Consultant Haematologist, Armed Forces Institute of Pathology (AFIP), Rawalpindi, Pakistan.; 3 Professor Saleem Ahmed Khan, FCPS, PhD, Professor of Pathology and Consultant Haematologist, Armed Forces Institute of Pathology (AFIP), Rawalpindi, Pakistan.

**Keywords:** Antithrombin III, Factor V Leiden, Protein C, Protein S, Thrombophilia

## Abstract

***Objectives:*** To determine the frequency of Protein C, Protein S (PC & PS), antithrombin deficiency (AT III) and Factor V Leiden mutation (FVL) as a cause of thrombophilia in the patients with venous thromboembolism (VTE) and cerebrovascular accident (CVA).

***Methods:*** It was an observational study conducted at Department of Haematology, Armed Forces Institute of Pathology (AFIP), Rawalpindi, Pakistan. All patients referred for thrombophilia screening from July 2009 to June 2012 were screened. Patients with evidence of VTE or CVA were screened for PC & PS, AT III deficiency, and FVL.

***Results:*** Total 404 patients of age between 1-71 years mean 33 ± 14 with male to female ratio of 2.4:1 had evidence of thrombophilia. Two hundred eighteen (54%) patients presented with CVA, 116 (29%) with deep vein thrombosis (DVT), 42 (10.5%) with pulmonary embolism (PE), and 28 (7.5%) with portal or mesenteric vein thrombosis (PV). Protein C & S deficiency was detected in 35/404 (8.7%), ATIII in 9/404 (2%), and FVL in 25/173 patients (14.5%). The findings were suggestive of a significant association of FVL mutation for developing DVT (OR=11.0, 95% C I 4.6-26.3), CVA (OR=5.7, 95% C I 2.1-15.1), and PV (OR=5.4, 95% C I 1.3-21.9). PC & PS deficiency was a significant risk factor for developing PE (OR=3, 95% C I 0.8-11.4).

***Conclusion:*** FVL mutation and Protein C & S are the leading causes of thrombophilia with strong association of Factor V Leiden mutation as risk for developing DVT.

## INTRODUCTION

Thrombophilia can be described as an abnormal predisposition to form clots within the vascular system. Normally the natural inhibitors of coagulation like Protein C (PC), Protein S (PS), antithrombin III (AT III) inhibit the coagulation factors activated during process of haemostasis. PC and PS destroys factor Va, and VIIIa to limit the coagulation process. A mutation in factor V gene (FVR506Q) translates Activated Protein C (APC) resistant factor V named as factor V Leiden (FVL). AT III forms complex with thrombin and to some extent with FIXa, FXIa, and FXIIIa to clear these factors rapidly from circulation. Deficiency of PC, PS, AT III or presence of FVL predisposes the individual to thrombophilia. In most of the cases the presence of inherited risk factor require interaction with other inherited or acquired risk factors before onset of a clinical disorder.^1^^-^^3^ When a person having genetic predisposition comes across a clinical setting associated with acquired thrombophilia, the risk of developing thrombosis increases as compared to normal population. Prevalence of hereditary thrombophilia (HT) in general population varies from 0.2%-0.4% for protein C deficiency, 0.2% for protein S deficiency, 0.02% for AT III deficiency and 4-5% for FVL.^4^^,^^5^

Thrombophilia screening for genetic risk factors is advised in patients with thromboembolism, particularly if they are young, present with recurrent episodes, thrombosis at unusual sites, or have a positive family history for the disease.^6^^,^^7^ Thrombophilia screening helps decision making about the duration of anticoagulant therapy after first episode, for instance a longer or indefinite duration of anticoagulant therapy.^8^ Whether the presence of genetic predisposition can predict the recurrence or not is much less clear due to conflicting results of various studies.^9^^,^^10^

The aim of this study was to determine the frequency of Protein C, protein S, AT III, and FVL mutation as a cause of thrombophilia in the patients with venous thromboembolism (VTE) and cerebrovascular accident (CVA). This study will enable us to recognize the association of most common genetic factors with the type of thrombophilia in our patient population.

## METHODS

It was an observational study conducted at department of Haematology, Armed Forces Institute of Pathology (AFIP), Rawalpindi, Pakistan. 

**Patients:** All patients referred for thrombophilia screening from July 2009 to June 2012 (total 404 patients) were screened. The study was approved by the Institute’s ethical committee for research, and all the procedures followed were in accordance with the ethical standards of the responsible committee on human experimentation and with the Helsinki Declaration of 1975, as revised in 2000. Patients with evidence of thromboembolism with following diagnostic criteria were included: 

***Deep vein thrombosis (DVT):*** Suggestive clinical history, and evidence of thromoembolism on duplex ultrasound.^11^

***Pulmonary embolism(PE):*** Suggestive clinical history, and evidence of thromboembolism on computed tomography (CT), pulmonary angiogram or on ventilation/perfusion (VQ) lung scans^11^

***Cerebro-vascular accident (CVA):*** Suggestive clinical history, neurological signs, and evidence on CT scan or Magnetic resonance imaging (MRI)^12^

***Portal vein thrombosis (PV):*** Suggestive clinical history, and evidence of thromboembolism on doppler-ultrasound, and/or computed tomography^13^

Patients on anticoagulant therapy or who had anticoagulant therapy within the last 4 weeks were excluded from study. Patients with liver disease, and recurrent pregnancy loss were also excluded. 

**Methods:** After recording demographic data, a brief clinical history and clinical examination blood samples were collected for ProC global test (PS & PC screening), AT III and FVL mutation screens by activated protein C (APC) resistance. Screening for FVL by APC resistance or molecular screening for FVL mutation was preformed only when requested by the treating physician. Molecular studies for prothrombin G20210A mutation, and screening for hyperhomocysteinemia were not performed due to lack of facilities. AT III assay was performed by automated coagulation analyzer (Sysmex CA-1500) using INNOVANCE antithrombin kit (Siemens Healthcare Diagnostics products GmbH). Antithrombin activity from 75-125% was taken as normal. Screening for PC, PS, and FVL was done using Pro C Global reagent kit by Siemens Healthcare Diagnostics products GmbH. In this assay incubation of test plasma with protein C activator and a contact phase activator causes activation of endogenous protein C and intrinsic coagulation cascade. Activated Protein C in conjunction with protein S inhibits factor VIIIa and Va.  This protein C activity dependant clotting time (PCAT) is in direct proportion to the Protein C and Protein S activity in test plasma. Normalized ratio of 0.69->1.56, and PCAT 85-200 seconds was taken as normal.   For FVL screening patient plasma was mixed with factor V deficient plasma in ratio of 1:4 and test was performed with same reagent kit as described for ProC global. Normalized ratio of 0.86-1.10 and PCAT 128-173 seconds was taken as normal. 

**Statistical analysis:** Data was collected on SPSS 19 and was analyzed for frequency by descriptive analysis, and statistical significance using Kruskal Walli’s test (for the comparison of more than two independent factors with dependant factor), a *p* value < 0.05 was taken as significant. The association of hereditary thrombophilia factors with the type of thromboembolism was established by calculating ODDs ratio (OR) with 95% CI

## RESULTS

Out of 2100 patients referred 404 patients between 1-71 years of age mean 33 + 14 had evidence of thrombophilia. Two hundred eighty eight (71%) were male and 116 (29%) were females with male to female ratio of 2.4:1. Seventy three percent of the patients had first episode, while 27% had second or repeated episodes without known risk factor. Majority of the patients had clinical features of thrombosis depending upon the anatomical site of thromboembolism; however 3% had associated bleeding manifestations also. Two hundred eighteen (54%) patients presented with CVA, 159(73%) were male and 59(27%) were female. One hundred sixteen patients (29%) had evidence of DVT, 74(64%) were male and 42(36%) were female. Forty two (10.5%) patients presented with PE, 35(83%) were male and 7(17%) were female. Twenty eight (7.5%) patients presented with PV, 20(71%) were male and 8(29%) were female. ProC global screening for PC & PS deficiency was suggestive of deficiency in 35/404 (8.7%). Antithrombin III deficiency was detected in 9/404 (2%) patients, and FVL in 25/173 patients (14.5%). Frequency of PC & PS, AT III deficiency and FVL in various disorders associated with HT is summarized in Table-I.

Factor V Leiden was a significant cause of CVA (*p*= < .001), DVT (*p*= < .001) and PV (*p*=.009). Antithrombin III deficiency was a significant cause of thrombophilia both in PV (*p*= < .001), and in CVA (*p*=.009). OR was suggestive of a significant association of FVL mutation with DVT (OR=11.0, 95% C I 4.6-26.3), CVA (OR=5.7, 95% C I 2.1-15.1), and with PV (OR=5.4, 95% C I 1.3-21.9). PC & PS deficiency was significantly associated with PE (OR=3, 95% C I 0.8-11.4). AT III deficiency was significantly associated with PV (OR=14.4, 95% C I 3.7-59.0).

**Table-I:** Frequency of PC & PS, AT III deficiency and FVL in various disorders associated with hereditary thrombophilia

**Disorder**	**PC & PS**	**AT III**	**FVL**
CVA	12/218 (5.5%)	2/218 (1%)	5/94 (5%)
DVT	16/116 (14%)	3/116 (2.6%)	17/51 (33%)
PE	5/42 (12%)	1/42 (2.4%)	2/22 (9%)
PV	2/28 (7%)	3/28 (11%)	1/6 (17%)
Total	35/404 (8.7%)	9/404 (2%)	25/173 (14.5%)

**CVA:** Cerebrovascular accidents

**PC & PS: **Protein C and Protein S.

**PE: **Pulmonary thromboembolism

**AT III: **Antithrombin III

**DVT: **Deep vein thrombosis

**FVL: **Factor V Leiden mutation.

**PV: **Portal/mesenteric vein thrombosis

## DISCUSSION

It is a well recognized fact that thrombophilia screening is advised more often than required due to serious nature of associated morbidity, though this association is not well established.^14^^-^^16^ The frequency of HT differs in various studies due to actual difference of frequency in geographical/ethnic groups, false positive/negative reporting, and the policy of various hospitals/physicians.^17^^,^ A false positive reporting is expected if thrombophilia screening is performed during the acute phase of episode, or patient had anticoagulant therapy within the last few weeks.^18^ The type of assay i.e. the immunological, functional or molecular is also important^19^, in our study we included patients with sufficient evidence of thromboembolism and chose functional assays only. 

Overall PC & PS deficiency was detected in 8.7% patients of unprovoked thrombophilia with 5.5% in CVA, 12% in PE, 14% in DVT, and 7% in PV. In a comprehensive study by Khalid et al. 2004 performed earlier in Pakistan, PC deficiency was detected in 2.3% and Protein S deficiency in 1.4%.^20^ In patients with adverse pregnancy outcome PC and PS deficiency was detected in 45% women that advocate the need of screening for natural anticoagulants while evaluating pregnancy loss.^21^ In meta analyses of CVA patients the frequency PC & PS deficiency has been reported as 14%, 19%, 23% in patients aged less than 45 years and 6% in patients aged less than 60 years.^22^ In a study by Jackson *et al* functional assay performed on 200,000 samples revealed a frequency of 13.7% for PC and 17.7% for PS, the difference in prevalence is probably real. ^23^

Antithrombin III deficiency was observed in 2% patients of throbophilia. It was detected 1% patients of CVA, 2.4% in PE, 2.6% in DVT and 11% in PV patients. In a previous study performed in same institute during 1996 overall frequency was 6.2% in patients <45 years of age. The difference is probably due to small sample size in previous study (n=32), and selection of younger age group.^24^ In study conducted by Khalid et al. frequency was 1.5%, however other international studies reported frequency of 5.5% -7.5%.^20^^,^^23^^,^^25^ Antithrombin III activity is affected by heparin therapy, if not considered prior to the assay and in patient on heparin therapy, false positive results are expected.

Overall FVL mutation was observed in 14.5% patients of thrombophilia. It was detected in 5% patients of CVA, 9% in PE, 33% in DVT and 17% in PV patients. In study conducted by Khalid et al. FVL mutation was detected in 14.2% patients that agree with current study.^20^ FVL mutation was detected in 1.3-4.3% of thrombophilia patients in earlier studies.^26^^,^^27^ There is marked heterogeneity in reported data about the frequency of FVL mutation in our population. In the study by Kreidy et al. 9.9% Lebanese patients with DVT were heterozygous for FVL mutation while in the study of Jackson et al., 17.7% had FVL mutation.^23^^,^^25^ It appears that FVL mutation may emerge as a leading cause of HT. 

## CONCLUSION

Factor V Leiden mutation, Protein C and S deficiency are the leading causes of thrombophilia with strong association of Factor V Leiden as a risk for developing DVT and CVA. Diagnostic workup of thrombophilia should include screening for FVL, protein C, protein S and AT III deficiency.

## RECOMMENDATIONS

1. Thrombophilia screening of all patients presenting with thromboembolism should be performed irrespective of age or site of thromboembolism.
2. Screening for Factor V Leiden mutation, PC, PS, and AT III deficiency should be included in thrombophilia screen.
